# Incarceration status at buprenorphine initiation and OUD treatment outcomes during pregnancy

**DOI:** 10.3389/fpsyt.2023.1157611

**Published:** 2023-04-13

**Authors:** Andrea Nguyen, Hannah Shadowen, Caroline Shadowen, Bhushan Thakkar, Andrea K. Knittel, Caitlin E. Martin

**Affiliations:** ^1^School of Medicine, Virginia Commonwealth University, Richmond, VA, United States; ^2^Department of Health Behavior and Policy, Virginia Commonwealth University, Richmond, VA, United States; ^3^Department of Obstetrics and Gynecology, Virginia Commonwealth University, Richmond, VA, United States; ^4^Department of Obstetrics and Gynecology, University of North Carolina at Chapel Hill, Chapel Hill, NC, United States; ^5^Institute for Drug and Alcohol Studies, Virginia Commonwealth University, Richmond, VA, United States

**Keywords:** opioid use disorder (OUD), perinatal, incarceration, buprenorphine, maternal health

## Abstract

**Introduction:**

Opioid use disorder (OUD) is a leading cause of pregnancy-associated deaths. OUD treatment with buprenorphine (BUP) reduces overdose risk and improves perinatal outcomes. Incarceration can be a barrier to receipt of OUD treatment during pregnancy and postpartum. The objective of this study was to examine differences in BUP continuation at delivery by patients’ incarceration status at the time of BUP initiation.

**Methods:**

This is a secondary analysis of a retrospective cohort study of pregnant patients with OUD who delivered at an academic medical center and initiated BUP between January 1, 2018, and March 30, 2020. The primary outcome was BUP continuation at delivery, abstracted from the state prescription monitoring program and electronic medical record, along with incarceration status. Bivariate analysis was used to assess the relationship between BUP continuation and incarceration status.

**Results:**

Our sample included 76 patients, with 62% of patients incarcerated at BUP initiation (*n* = 47). Among the entire sample, 90.7% (*n* = 68) received BUP at delivery. Among patients who were incarcerated at BUP initiation, 97% remained on BUP at delivery; among patients who were not incarcerated at BUP initiation, 79% remained on BUP at delivery (*p* = 0.02).

**Conclusion:**

In our sample from a health system housing a care model for pregnant and parenting people with OUD with local jail outreach, BUP continuation rates at delivery were high, both for patients who were and were not incarcerated at BUP initiation. Findings are intended to inform future work to develop and evaluate evidence-based, patient-centered interventions to expand OUD treatment access for incarcerated communities.

## 1. Introduction

In the United States, opioid use disorder (OUD) is a leading cause of pregnancy-associated deaths (1). Medications for OUD (MOUD), including buprenorphine (BUP), reduce overdose risk and improve perinatal outcomes (2). One important factor influencing OUD treatment continuation is the involvement of the criminal legal system (3). SAMHSA recommends MOUD be offered to all people with OUD during incarceration (4). However, several barriers to MOUD provision during incarceration and its transitions pre/post-release exist, such as inconsistencies across states in insurance coverage (e.g., Medicaid not accessible during incarceration) and levels of access to medical specialty services across institutions (e.g., carceral systems are fiscally responsible for medical care) (5, 6).

Receipt of medications for OUD during incarceration is rare, including during pregnancy and postpartum; Sufrin et al. (7) recently reported that nearly one third of pregnant people with OUD entering to prisons and jails were either withdrawn from treatment or not offered MOUD while withdrawing from opioids. This is unfortunate, as provision of MOUD during incarceration can promote positive social outcomes, such as decreased recidivism (8), decreased mortality post-release (9), and better community engagement (10). Other than high rates of MOUD discontinuation occurring postpartum in jails and prisons, little is known regarding OUD treatment outcomes among incarcerated pregnant individuals.

Nonetheless, innovative models of care are emerging to address these significant unmet treatment needs among people who are incarcerated, including during the highly vulnerable life-course periods of pregnancy and postpartum. For example, a recent study done in North Carolina highlighted the potential of a prison-academic partnership to bolster MOUD continuity for pregnant and postpartum people with OUD (11). Likewise, our institution houses an integrated OBGYN-Addiction program consisting of nurses and medical providers with expertise in both OBGYN and Addiction Medicine as well as support staff, behavioral health clinicians and subspecialty consultants who provide robust, recovery-oriented wrap-around services (12). An integral component of this program includes its partnerships with local jails where pregnant individuals with OUD are referred to our health system for evaluation and initiation of OUD treatment. Specifically, pregnant people who present with opioid withdrawal at incarceration are transported to our OBGYN antepartum service for evaluation and are offered BUP initiation while inpatient; before discharge, outpatient follow-up is coordinated by nursing staff with the local jail and the OBGYN-Addiction program.

This partnership offers an opportunity to evaluate OUD treatment outcomes among this vulnerable, highly understudied population. The primary objective of this study is to compare BUP continuation rates until delivery by incarceration status at BUP initiation among a cohort of pregnant patients with OUD seen within our health system. In doing so, we discuss our findings in the context of the clinical practices that our innovative integrated OBGYN-Addiction care model for pregnant and parenting people utilizes to expand its reach to local incarcerated individuals with OUD.

## 2. Methods

### 2.1. Design

The current study is a secondary analysis of a retrospective cohort study exploring health and addiction outcomes for pregnant and postpartum patients who received buprenorphine for OUD at Virginia Commonwealth University (VCU), an academic medical center. Briefly, electronic medical record of patients receiving BUP (sublingual tabs or films, buprenorphine or buprenorphine-naloxone) at any point during pregnancy and/or through 1 year postpartum from January 2017 to March 2020 were included. Detailed methods for the parent study are described elsewhere (13). This academic medical center has a designated OBGYN-Addiction clinic staffed by Obstetricians that provides integrated care, behavioral and medical care for many of the individuals in this study. While receiving care at this clinic was not a requirement of study participation, many individuals did receive care in this clinic. A study team performed a manual abstraction of the electronic medical record, which included review of buprenorphine prescriptions documented by the Virginia Prescription Monitoring Program. Chart abstractions were done in 4-week increments during the perinatal period for clinical and psychosocial data, including pregnancy outcomes, incarceration status and OUD treatment outcomes. Incarceration status was ascertained from provider documentation. Chart abstractions started at the time of initial BUP receipt during pregnancy and continued until delivery. The larger study was done with IRB approval from Virginia Commonwealth University.

### 2.2. Participants

**FIGURE 1:**
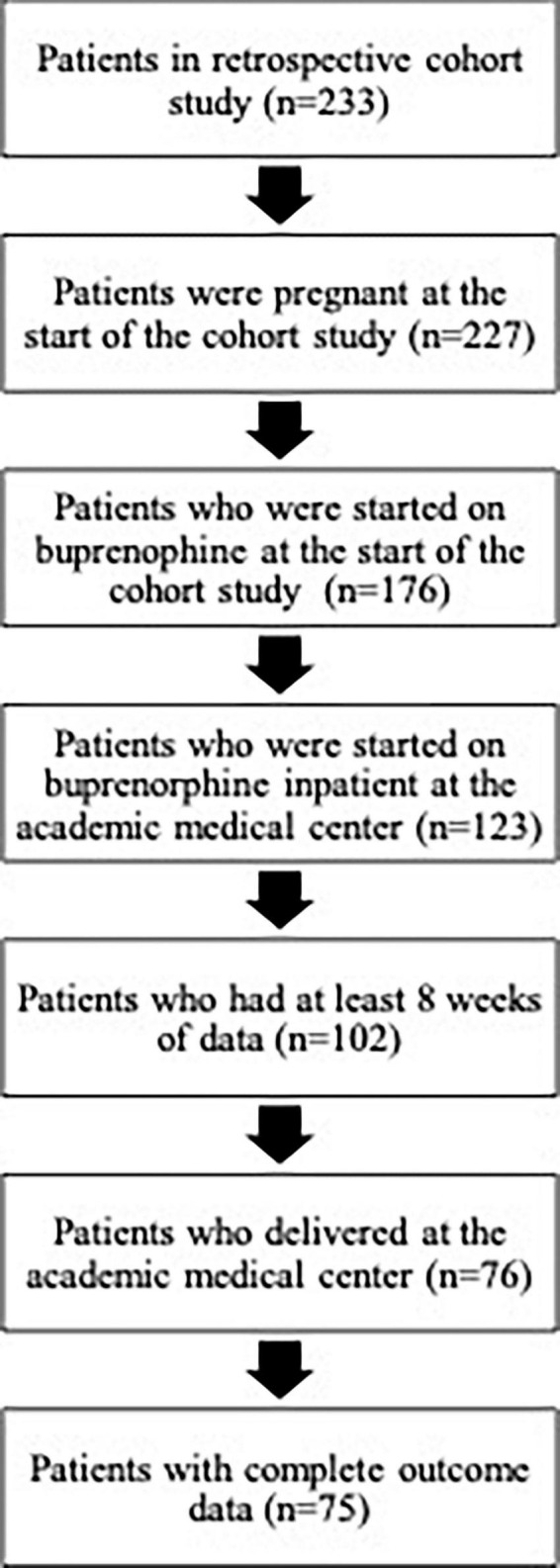
Flowchart of obtaining study sample from the parent retrospective cohort study through exclusion criteria.

Patients were included in the current secondary analysis if pregnant at the time of BUP initiation, had at least 8 weeks of longitudinal data (with complete outcome ascertainment), delivered at VCU, and started BUP while inpatient at the study institution. See Figure 1 for more details.

### 2.3. Analytic plan

To evaluate differences in demographic and clinical variables of patients who were incarcerated at BUP initiation versus patients who were not incarcerated, we used chi-square tests and student *t*-tests. Next, we again used chi-squared and *t*-tests to examine the relationship between our primary outcome, BUP continuation at delivery, and our main exposure variable of interest, incarceration status at BUP initiation, within our final sample. All analysis was done with STATA 17 (14).

## 3. Results

Our study included 75 individuals (Table 1). Most of our sample (62.6%) was incarcerated at BUP initiation, enrolled in Medicaid (69.3%), identified as white (73.3%), and 30.7% had a high school diploma or GED. The median dosage of BUP after inpatient BUP induction was 12 mg daily (range 2, 24). Individuals who started BUP while incarcerated were less likely to have current Medicaid coverage than their non-incarcerated counterparts (61.7% vs. 82.1%; *p*-value = 0.045), as documented at the time of buprenorphine initiation.

**TABLE 1:** Demographic and clinical variables of patients in study sample, by incarceration status at buprenorphine (BUP) initiation**.

	Total (*n* = 75)	Not incarcerated at BUP* initiation (*n* = 28)	Incarcerated at BUP initiation (*n* = 47)	*p*-value
Age (mean; std)	28.9 ± 4.4	28.5 ± 3.5	29.2 ± 4.9	0.47
Education				0.299
Less than high school diploma	10 (13.3)	7 (25.0)	3 (6.4)	
High school diploma/GED	23 (30.7)	11 (39.3)	12 (25.5)	
College education	9 (12.0)	3 (10.7)	6 (12.8)	
Not reported	33 (44.0)	7 (25.0)	26 (55.3)	
Race^†^				0.001
Black or African American	19 (25.3)	13 (46.4)	6 (12.8)	
White	55 (73.3)	15 (53.6)	40 (85.1)	
Not reported	1 (1.3)	0 (0.0)	1 (2.1)	
Insurance^‡^				0.045
Medicaid	52 (69.3)	23 (82.1)	29 (61.7)	
Private	3 (4.0)	2 (7.1)	1 (2.1)	
None	12 (16.0)	1 (3.6)	11 (23.4)	
Other	8 (10.6)	2 (7.1)	6 (12.8)	
Comorbid mental health conditions^§^				0.591
No	27 (36.0)	9 (32.1)	18 (38.3)	
Yes	48 (64.0)	19 (67.9)	29 (61.7)	
Family history of substance use disorder				0.991
No	27 (36.0)	13 (46.4)	14 (29.8)	
Yes	25 (33.3)	12 (42.9)	13 (27.7)	
Not reported	23 (30.7)	3 (10.7)	20 (42.6)	
Co-occurring substance use disorder^||^				0.050
No	43 (57.3)	12 (42.9)	31 (66.0)	
Yes	32 (42.7)	16 (57.1)	16 (34.0)	
Estimated gestational age at delivery (median; range) ^¶^	39 (23, 41)	38 (23, 41)	39 (30, 41)	0.857
Dose of BUP at discharge from inpatient BUP initiation (median; range) ^¶^	12 (1, 24)	12 (2, 24)	10 (2, 24)	0.691
Incarcerated at delivery				<0.001
No	45 (60)	26 (92.9)	19 (40.4)	
Yes	30 (40)	2 (7.1)	28 (59.6)	
Continued BUP until delivery				0.050
Yes	68 (90.7)	23 (82.1)	45 (95.7)	
No	7 (9.3)	5 (17.9)	2 (4.3)	

Data are *n* (%). Significant at *p*-value < 0.05.

*BUP, buprenorphine.

**Excludes not reported observation in chisquared tests.

^†^Self-reported race by patient as documented in medical record. Identifiers include Native American or Alaska Native, Asian, Black or African American, Native Hawaiian or other, white, Hispanic, not reported. Only included categories that individuals identified with in the table.

^‡^Insurance information was abstracted upon initial encounter. For those incarcerated, if they were seen outpatient initially, they were charted as having jail insurance (noted in the “other” insurance category). However, if they were seen inpatient initially, they remain on Medicaid and were thus charted as having Medicaid.

^§^Conditions include ADD/ADHD, anxiety, bipolar/mania, depression, schizophrenia, PTSD, other.

^||^Co-occurring substance use disorders include cocaine, benzodiazepine, cannabis, amphetamine.

^¶^Non-parametric equality of means test used to assess differences between those who were incarcerated at BUP initiation and those who were not.

Regarding BUP continuation during pregnancy, most individuals (90.7%; *n* = 68) in our sample remained on BUP at delivery. The proportion continuing BUP until delivery was slightly higher among individuals who were incarcerated at time of BUP initiation compared to individuals who were not incarcerated (95.7% vs. 82.1%; *p*-value = 0.05).

## 4. Discussion

Within our sample, results demonstrate similarly high BUP continuation rates at delivery regardless of incarceration status at BUP initiation during pregnancy. These findings are encouraging, as incarceration is typically a barrier to OUD treatment. At our institution, we developed a partnership with local jails where pregnant individuals who present with opioid withdrawal at incarceration are referred to our hospital antepartum service for evaluation and initiation of BUP with outpatient follow-up after discharge. We postulate that this OBGYN-Addiction care model may have contributed to our positive findings.

Incarceration-based MOUD programs can positively impact health outcomes. A recent study interviewed jail representatives across the United States to evaluate available resources and practices. Authors report that 96% of jails have a physician-approved protocol to address opioid withdrawal; however, fewer (81%) use an FDA-approved medication for withdrawal management (15). A study in England found MOUD provision in prisons was associated with a 75% reduction in all-cause mortality and an 85% reduction in fatal drug-related poisoning in participants’ first months post-release (9). Similarly, in Rhode Island, no study participants who started BUP while incarcerated experienced an overdose after release nor reported any opioid use recurrence 6 months post-release (16). Our study results extend the findings of these studies into the perinatal period, overall demonstrating the important role that incarceration based MOUD programs could play in reducing morbidity and mortality due to OUD throughout transitions in and out of incarceration.

Common models of MOUD provision for incarcerated people include: jail/prison staff transporting patients to clinic, jail/prison staff themselves picking up medications to bring back to patients, and integrated clinics within jails/prisons (17). The program embedded within the health system from which this study derives provides outpatient substance use disorder treatment integrated with OBGYN care. As part of this program, providers travel to one local jail to provide care on site, and other jails transport pregnant patients on BUP to the health system outpatient OBGYN clinic approximately monthly for OUD treatment follow-up while incarcerated during pregnancy (12). Recently, due to the COVID-19 pandemic, some jails and prisons have implemented telemedicine to provide further flexibility for MOUD (18). While we found high rates of BUP continuation at delivery for incarcerated patients, it is important that this finding not be interpreted as a recommendation for incarceration as an addiction treatment modality. Carceral systems are fiscally responsible for the healthcare of all inmates and thus may coincidentally serve as an opportunity for MOUD. Our findings highlight how harnessing this opportunity may be enhanced *via* community or academic partnerships. Overall, more research is needed to evaluate outcomes for various methods of delivering MOUD to incarcerated people to optimize OUD care and inform policies impacting this vulnerable population.

Despite the recommendation that MOUD be provided to all patients with OUD regardless of incarceration status, actual MOUD provision varies widely between incarceration facilities (15). Legislation is greatly needed to standardize this care (5). Interruptions in Medicaid coverage between incarceration and release likely contribute to this important public health issue (6). Notably, many incarcerated patients in our sample were without Medicaid coverage at the time of BUP initiation, reflecting missed opportunities for these individuals to gain coverage in our Medicaid expanded state. Such interruptions in OUD treatment increase the risk for recurrence of use and other adverse outcomes, as patients may be at high risk of overdose when released from incarceration without continuity in MOUD provision. Legislation to cover immediate Medicaid coverage upon reentry, or even ongoing coverage during incarceration, could potentially prevent such gaps in care and facilitate continuity of MOUD treatment. Additionally, the provision of MOUD to incarcerated persons could, in turn, increase treatment engagement in the community upon release, ultimately improving health and psychosocial outcomes (19).

The goal of this study was to examine BUP continuation rates at delivery in pregnant patients who initiated BUP while incarcerated versus not incarcerated. Our results in the context of this existing literature support that, while incarceration is not a recommended addiction treatment pathway, incarceration can serve as an important entry point for OUD care during pregnancy. Additionally, carceral-academic partnerships, in some settings, may improve continuity of care for pregnant and parenting people with OUD. Study limitations include information bias due to the use of the medical records as the data source, rather than primary data collection. Additionally, the patient perspective of treatment and the OBGYN-Addiction program partnership was not evaluated, an area for future investigation. The replicability of the results are unknown due to the small sample and are unadjusted, so results should be interpreted as preliminary. At our institution, pregnant individuals who are transported from local jails must be admitted to the hospital for inpatient observation for BUP initiation. We recognize that this may have generated a sampling bias and may not be a feasible option for other healthcare centers.

Study findings suggest that pregnant individuals receiving MOUD can achieve similar treatment outcomes regardless of incarceration status. The incorporation of an incarceration-based MOUD program partnered with an OBGYN-Addiction program affiliated with an academic health system is feasible and potentially shows preliminary effectiveness at increasing use of life-saving treatments for pregnant individuals seeking OUD recovery. Ultimately, further work is needed to expand access and MOUD continuity for pregnant and postpartum individuals experiencing incarceration. Future studies should evaluate different modes of BUP utilization for incarcerated people to investigate how incarceration status impacts OUD treatment trajectories for this unique patient population.

## Data availability statement

The raw data supporting the conclusions of this article will be made available by the authors, without undue reservation.

## Ethics statement

The studies involving human participants were reviewed and approved by the Virginia Commonwealth University IRB. The patients/participants provided their written informed consent to participate in this study.

## Author contributions

AN: conceptualization, writing – original draft, and writing – review and editing. HS: data curation, formal analysis, and writing – review and editing. CS: conceptualization and writing – review and editing. BT: conceptualization, methodology, and writing – review and editing. AK: visualization and writing – review and editing. CM: conceptualization, methodology, project administration, supervision, writing – review and editing, and funding acquisition. All authors contributed to the article and approved the submitted version.
